# Regulatory T Cell-Derived Exosomes: Possible Therapeutic and Diagnostic Tools in Transplantation

**DOI:** 10.3389/fimmu.2014.00555

**Published:** 2014-11-05

**Authors:** Akansha Agarwal, Giorgia Fanelli, Marilena Letizia, Sim Lai Tung, Dominic Boardman, Robert Lechler, Giovanna Lombardi, Lesley A. Smyth

**Affiliations:** ^1^Medical Research Council (MRC) Centre for Transplantation, King’s College London, Guy’s Hospital, London, UK

**Keywords:** regulatory T cells, exosomes and immune modulation

## Abstract

Exosomes are extracellular vesicles released by many cells of the body. These small vesicles play an important part in intercellular communication both in the local environment and systemically, facilitating in the transfer of proteins, cytokines as well as miRNA between cells. The observation that exosomes isolated from immune cells such as dendritic cells (DCs) modulate the immune response has paved the way for these structures to be considered as potential immunotherapeutic reagents. Indeed, clinical trials using DC derived exosomes to facilitate immune responses to specific cancer antigens are now underway. Exosomes can also have a negative effect on the immune response and exosomes isolated from regulatory T cells (Tregs) and other subsets of T cells have been shown to have immune suppressive capacities. Here, we review what is currently known about Treg derived exosomes and their contribution to immune regulation, as well as highlighting their possible therapeutic potential for preventing graft rejection, and use as diagnostic tools to assess transplant outcome.

## Treg Exosomes – Immune Modulators

Exosomes are small, cup-shaped, secreted membrane vesicles (approximately 50–100 nM in diameter) that are formed by the inward budding of endosomal membranes (1–6). Exosomes are released into the extracellular environment following the fusion of multivesicular endosomes with the plasma membrane (7). Several proteins involved in their biogenesis and release have been described and have recently been reviewed by Colombo et al. (7). Exosomes released by many immune and non-immune cells have been shown to have a range of physiological properties within the immune system. These include antigen presentation, immune regulation, and programed cell death, each of which is linked to the cell from which they are released (6, 7). They play an important role in intercellular communication and can act as shuttles for transferring proteins, miRNA, mRNA, and cytokines from one cell to another (8).

Many cells of the body produce these extracellular vesicles (EVs) including those of the immune system such as CD4^+^ and CD8^+^ T cells, B cells, and dendritic cells (DCs). Exosomes from these cells have been shown to mediate either immune stimulation (DCs) or immune modulation (T cells) (9–14). Recently, the release of exosomes by murine CD4^+^CD25^+^Foxp3^+^ regulatory T cells (Tregs), following TCR activation, was shown, initially by Smyth et al. (15) and later by Okoye et al. (16). In addition to CD4^+^CD25^+^Foxp3^+^ cells, other murine T cells with regulatory capacities were found to also release exosomes following activation. Bryniarski et al. observed that “exosome like” particles were present in the supernatants of cultured CD8^+^ T cells with suppressive capacity (17), whilst Xie et al. observed that CD8^+^CD25^+^Foxp3^+^ T cells secreted exosomes capable of inhibiting DC induced CD8^+^ CTL responses (18).

Exosome production by murine CD4^+^CD25^+^Foxp3^+^ Tregs appears to be quantitatively greater than other murine T cells, including naïve CD4^+^ and CD8^+^ T cells, T helper 1 (Th1), and Th17 cells, and is regulated by changes in intracellular calcium, hypoxia, and sphingolipids ceramide synthesis, as well as in the presence of IL-2 (16). Exosomes contribute significantly to the function of murine CD4^+^CD25^+^FoxP3^+^ Tregs, inhibiting the release of exosomes reversed these cells suppressive capabilities (16). In parallel, murine Tregs exosomes were found to be immune modulatory. Reduced CD4^+^ T cell proliferation and cytokine (IL-2 and IFNγ) release was observed in their presence *in vitro* (15). The suppressive nature of Treg exosomes, in one study, has been attributed to the ectoenzyme CD73 (15). The loss of CD73 on Treg exosomes reversed their suppressive nature. Expression of both CD39 and CD73 on Tregs contributes to immune suppression through the production of the anti-inflammatory mediator adenosine (19–21). Binding of this molecule to adenosine receptors A2aR, expressed by activated T effector cells (Teffs) triggers intracellular cAMP leading to the inhibition of cytokine production, thereby limiting T cell responses (22). Given that adenosine was produced following incubation of CD73 expressing Treg exosomes with exogenous 5′AMP it is feasible that the release of exosomes expressing CD73 within the local environment increases the surface area by which this membrane-associated enzyme, and ultimately Treg suppression, can function (15).

Several molecules associated with immune modulation including CD25 and CTLA-4, were also found on CD4^+^CD25^+^Foxp3^+^ Treg exosomes (15). Nolte-’t Hoen et al. have previously shown that exosomes, derived from anergic rat T cells, inhibited Teffs responses following co-culture with B cells and DCs *in vitro* (23). These T cell-derived exosomes expressed high levels of CD25 and the authors suggested that CD25 expressing exosomes, binding to the surface of an antigen presenting cells (APC), bestows that cell with the ability to bind free IL-2 in the local environment leading to depletion of available cytokines and apoptosis of Teffs (23). Although CD25 expression was observed on Treg exosomes, this molecule may not play a role in their suppressive function given the observation that exosomes isolated from a T cell line, incapable of suppressing proliferation or cytokine production of CD4^+^ T cells, in the presence of B cells, expressed similar levels of CD25 to Treg exosomes with regulatory function (15). A redundant role for CTLA-4 molecules has also been reported. Although present on Treg exosomes, blocking CTLA-4 did not modulate their suppressive function (15). So far, no molecules have been associated with the regulatory capacity of CD8^+^25^+^FoxP3^+^ exosomes (18).

Recently, the transfer of miRNAs contained in T cell exosomes has been shown to affect the function of recipient APCs by inhibiting translation of target mRNA molecules (14, 24). Likewise, the transfer of miRNAs, including Let-7d, miR-155, and Let-7b, to Teffs through the acquisition of CD4^+^CD25^+^Foxp3^+^ Treg exosomes has been shown (16). Inhibiting Let-7d expression in Treg exosomes reversed the suppressive nature of these vesicles suggesting that miRNAs present in Treg exosomes may also play a role in their suppressive capacity (16). These findings confirm those of Bryniarski et al. (17) who observed the targeted delivery of an inhibitory miRNA, miR-150, to Teffs using exosomes isolated from CD8^+^ T cells with suppressive capacity.

Several molecules present on exosomes isolated from Teffs, DCs, and B cells have been shown to have immune modulatory properties. Whether they also contribute to the suppressive nature of Treg exosomes has yet to be validated. For example, expression of FasL on murine CD8^+^ T cell exosomes induced death of APCs (12, 25), in addition, FasL-expressing exosomes isolated from DCs, genetically modified to express FasL, suppressed antigen-specific immune responses *in vivo* (26) and lastly, MHCII^+^FasL^+^ exosomes constitutively produced by a human B cell-derived lymphoblastoid cell lines induced apoptosis in CD4^+^ T cells (27). Murine and human CD4^+^25^+^ Tregs express FasL (28). Whether FasL is expressed on Treg exosomes and contributes to the death of Teffs is yet to be tested. Other molecules, present on Tregs such as the inhibitory cell surface ligand programed cell death 1 ligand 1 (PDL-1) and Galectin-1 (29–31) may also be present on Treg exosomes. PDL-1 was found on mesenchymal stem cell EVs (32) and exosomes have been identified as transport vehicles for the secretion of molecules that lack a signal sequence such as Galectin-1 (33). Not only is this molecule highly expressed on Tregs it is essential for their function (34).

Regulatory T cells produce immune modulating cytokines such as IL-10, IL-35, and TGFβ (35). Presently, it is unknown whether these cytokines are contained in Treg exosomes however, expression of IL-10 and TGFβ in exosomes isolated from DCs, transduced to express these cytokines, has been shown (36, 37) as has surface TGFβ on MSC derived EVs (32). Given the aforementioned it is a theoretical possibility, that Treg exosomes may contain one or more of these cytokines.

## Role of Treg Exosomes in Transplantation

### Possible therapy?

In 1990, Hall et al. observed that the adoptive transfer of CD4^+^CD25^+^ T cells resulted in long-term cardiac allograft survival in cyclosporine-treated rats (38). Since then this field of immunotherapy has been intensely studied in mouse (39–41), and recently in preclinical humanized mouse models (mice reconstituted with a human immune system and transplanted with human skin or human pancreatic islets of Langerhans) (42, 43). In the latter, human CD4^+^25^+^Tregs, expanded with anti-CD3/28 antibody coated beads, have been found to prolong islet transplant survival and function (42, 44). These positive outcomes have led to the application of humans Tregs for the prevention of graft versus host disease (GvHD) and to promote transplant tolerance (45–48). Currently, several organizations around the world are investigating the use of CD4^+^CD25^+^ Tregs to promote “tolerance” to transplanted organs. At King’s College London, UK, phase I/II clinical trials are currently under way to test the safety and efficacy of using these cells in human kidney (One Study) and liver (ThRIL) transplant patients. Other clinical trials using human Tregs are also underway and are described elsewhere (49). Presently, we do not know the efficacy and efficiency of Tregs in these trials. Although Tregs are now being used in patients how they function *in vivo* is still unknown.

**Figure 1:**
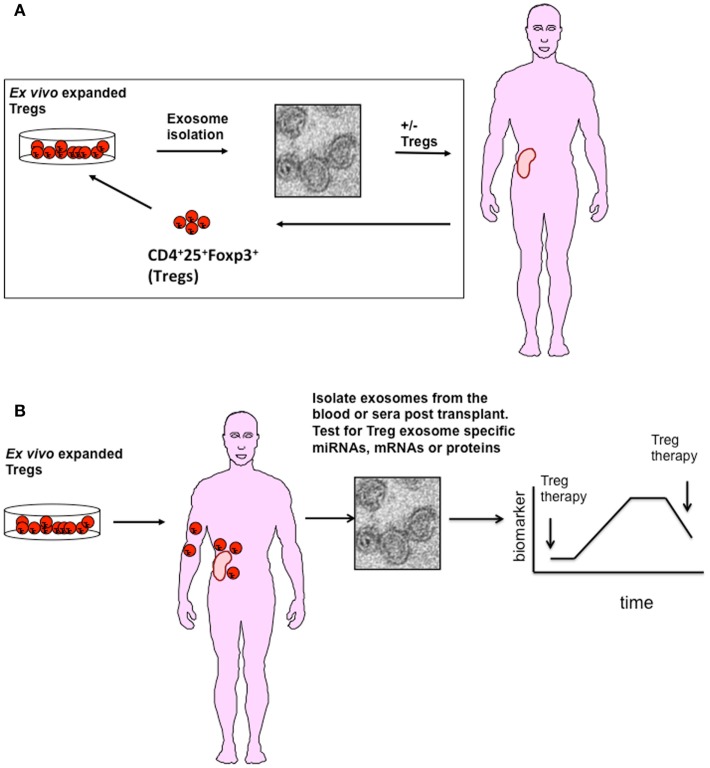
**A possible role for Treg exosome in transplantation**. **(A)** Exosomes isolated from *ex vivo* expanded polyclonal or antigen-specific Tregs represent a potential adoptive therapy tool to promote transplant tolerance. Exosomes isolated from activated Tregs either alone or modified to express specific inhibitory miRNAs, chemicals, or cell surface molecules could be used in conjunction with Tregs to promote transplant tolerance. **(B)** Following transplantation, exosomes released from Tregs maybe used as a diagnostic tool to monitor activation and survival of Tregs *in vivo*. As Tregs release exosomes following activation, interaction with APCs expressing alloantigen on grafted tissue will result in exosome release. Identifying specific miRNAs expressed in Treg exosomes will help in their identification in blood or urine.

Given their immune modulatory capacity, the question arises, what is the contribution of Treg exosomes to transplant tolerance seen in the preclinical mouse models and can Treg exosomes be used *in vivo* as an alternative/or complementary therapy? At present, we are a long way away from using Treg exosomes in man given that the optimal Treg subset required to induce transplant tolerance is as yet unknown, as is whether they prolong graft survival in a patient setting. So why should we consider these EVs as a therapy? Several studies have suggested that inflammatory environments can subvert human Foxp3^+^Treg cell function by converting them to Teffs *in vivo* (50, 51). However, unlike Foxp3^+^ Tregs, adoptive transfer of human Treg exosomes are unlikely to be modified during inflammatory conditions *in vivo* (1) making them an ideal immune modulatory reagent (Figure 1A).

Several lines of evidence exist, some preliminary, some not, suggesting that studying these vesicles for this purpose is worthwhile, albeit challenging. So far, Yu et al. are the only group that have investigated the use of Treg exosomes as a therapy in a transplantation setting (52). These authors observed that the adoptive transfer of autologous rat Treg exosomes, post transplant, prolonged both survival and function of kidney allografts (52). Suggesting that Treg exosomes may represent an exciting new therapy for the induction of transplant tolerance.

Can this observation be translated into a human setting? Using preclinical methods to isolate and expand human Tregs, from peripheral blood of health individuals (53), we have successfully identified the release of CD63 and CD81 expressing exosomes from CD4^+^CD25^hi^Foxp3^+^ suppressive human Tregs, following TCR activation (Agarwal et al., personal communication). Whether human Treg exosomes display molecules that can modulate the immune response *in vivo* is still being assessed. However, given that Jurkat CD4^+^ T cells (a human T cell line) as well as human CD3^+^ T cells, isolated from PBMCs, produce exosomes (54–56) containing molecules with potential immune regulatory effects, such as TCRs (54) and CTLA-4 (56) the possibility that human Treg exosomes contain immune regulatory molecules is very high.

Two phase I clinical trials using exosomes isolated from immature DCs have been conducted in advanced stage melanoma and MAGE-expressing non-small cell lung cancer patients (57–59). Despite a lack of antigen-specific T cell responses, stable disease was observed in some patients with tumor regression reported in one patient following treatment (60–62). These positive outcomes have paved the way for Phase II clinical trials using exosomes isolated from LPS or IFNγ activated DCs in non-small cell lung cancers. These studies have validated the efficacy and safety of exosomes as a therapy in man. In spite of these encouraging findings, several key limitations pertaining to the use of exosomes cannot be ignored. Firstly, at present there is no standardized protocol for isolating and analyzing “pure” exosomes (7). Contamination from other EVs as well as membrane free aggregates may be an issue depending on the isolation method used. Therefore, careful analyses of the purified exosomes will be required before administration. This will require the use of expensive equipment such as EM and Nanosight, which are not always readily available (7, 63). Secondly, given that exosome release by Tregs is not constitutive and requires activation using anti-CD3/CD28 antibodies, the possibility that these antibodies contaminate Treg exosome preparations is as yet untested. Additionally contaminating molecules, for example, proteins/cytokines present in media, may pose a potential problem especially as exosomes will be isolated from culture supernatants. Thirdly, the quantity of exosomes isolated and the amount required for therapy purposes are at present unknown, as is whether large-scale production of Treg exosomes is actually possible. Lamparski et al. published that 1.8–5.8 mg of exosomes could be isolated from human monocyte derived DCs, expanded from peripheral blood leukopacks (originally containing 12–25 × 10^9^ cells) higlighting the feasibility of large-scale production of DC exosomes (64). However, DCs produce these EVs vesicles constitutively making their production easier than those from Tregs, which are isolated only after activation (65). Yu et al. obtained 117 μg of exosomes from 4 × 10^9^ freshly isolated rat Tregs, following activation, and the administration of 33 μg of exosomes, given over 3 time points, was sufficient to prolong the lifespan of a kidney transplant (52). Whether large quantities of pure exosomes can be isolated from human Tregs grown under GMP conditions is as yet unknown. Lastly, what happens to Treg exosomes *in vivo*, which cells acquire them and whether is it receptor driven is poorly understood. Recently, Teffs were shown to acquire Treg exosomes (16) whilst exosomes from EL4, a T cell lymphoma, have been shown to be preferential acquired by macrophages (66), perhaps via the CD169 pathway (67). Therefore, *in vivo* analysis of Treg exosomes is essential before they can be used in a clinical setting. Until all of these factors are addressed, using Treg exosomes in a transplant setting remains challenging and potential advantages remain at present theoretical.

### Diagnostic tool?

Biomarkers are quantitatively, measurable biological parameters that help indicate health and disease. The use of exosomes as biomarkers is a relatively new concept. Although it has not yet reached clinical practice, it is one area of exosome research that is rapidly expanding, with many clinical trials focusing on their use as a diagnostic tool, particularly for cancer (Table 1). Several factors make exosomes suitable for this purpose, firstly, they travel through the bloodstream and can be isolated from plasma, serum, and urine (68, 69). Secondly they receive surface markers from the cell from which they are derived, such that they can be identified and isolated. Lastly, they express unique miRNA and mRNA (Table 1).

**Table 1:** **miRNAs present in exosomes isolated from the sera of patients with specific cancers or following immunization are being used as diagnostic biomarkers**.

miRNA identified in exosomes	Cells origin	Reference
miR-150	CD4^+^ T cells	(70)
miR-21, miR-141, miR-200a, miR-200b, miR-200c, miR-203, miR-205, and miR-214	Ovarian cancer	(71)
miR-205, miR-19a, miR-19b, miR-30b, and miR-20a	Lung squamous cell carcinoma	(72)
let-7a, miR-1229, miR-1246, miR-150, miR-21, miR-223, and miR-23a	Colon cancer	(73)
hsa-miR-31, miR-185, and miR-34b	Melanoma	(44)

Valadi et al. were the first group to publish that exosomes contained RNA (8). Exosome RNA is small, typically of about 200 bases in length and lacks the 18S and 28S RNA found in cells (74). Different RNA species including small ribosomal RNA, specific tRNA fragments, long interspersed elements, and long terminal repeats, have all been found in exosomes (75). Additionally, and as discussed earlier, there is also a selective enrichment of specific miRNAs into exosomes (24, 76). The miRNA repertoire of an exosome is generally different to that of the parent cell, suggesting that exosome packaging is an active process (14). In T cells, for example, Rossi et al. identified a set of 20 miRNAs of which only 2 were differentially expressed in T_H_ cell-derived exosomes (77). Upon activation primary CD4^+^ T cells down-regulate their miRNA content. Some of these miRNAs accumulate in exosomes, for example, miR-150, suggesting that the cell may be shedding miRNA as part of a regulation step (70). de Candia et al. quantified the amount of miR-150 present in sera isolated from mice immunized with OVA plus an adjuvant, and reported an increased level of this miRNA in immunized mice as compared to non-immunized mice (70, 78). When they removed CD4^+^ T cells no elevated miR-150 levels were observed. They next validated this observation using sera collected from adults and children vaccinated with the 2009 pandemic flu (H1N1) vaccine. Similar to the mouse model, they observed that miR-150 was evident in the sera following vaccination, and that this miRNA was associated with lymphocyte derived exosomes. In addition, increased levels of miR-150 correlated with high antibody levels post vaccine, suggesting a link between activation of the adaptive immune responses and expression of a specific miRNAs in exosomes (70, 78). From the adoptive cellular therapy point of view, this data is very exciting as it highlights the possibility of using exosomes to monitor cellular therapies such as Tregs *in vivo*. Given that Tregs produce exosomes only following activation, and in the case of transplantation this will be following recognition of alloantigen presented by donor and recipient DCs, it may be possible to assess Treg viability and function *in vivo* by monitoring Treg exosomes in the blood of transplant recipients. If this is possible Treg exosomes may be unique biomarkers for immune suppression (Figure 1B).

As mentioned earlier in addition to miRNA, mRNA, and proteins associated with exosomes can also act as diagnostic tools. For example, in patients with kidney disease CD2AP mRNA was associated with urinary exosomes (79). Several specific proteins have been identified in exosomes isolated from: (1) the urine of healthy individuals (CD24 and Aquaporin 2) (80), (2) sera from cancer patients (MUC1, LRG1, Hsp90a, and RAD21) (81), (3) the placenta (syncytin-1) (82), and 4) from patients with multiple sclerosis (IB4) (83). Taken together, these studies suggest the importance of validating the expression of mRNA and proteins, in addition to miRNAs, in Treg exosomes if unique biomarkers are to be identified.

In conclusion, at present Treg exosomes are still in their infancy with regard to transplantation, either as a therapy or a diagnostic tool. As outlined in this review, several key questions regarding their composition and function need to be addressed. In addition, better isolation and analysis protocols, as well as preclinical models are required before Treg exosomes can make the transition from the lab to the clinic, even for diagnostic purposes. Although some of the ideas presented here are speculative, pursuing the use of Treg exosomes for immune modulation and diagnostic purposes within a transplantation setting is timely given that clinical trials are now underway using Treg cells themselves.

## Conflict of Interest Statement

The authors declare that the research was conducted in the absence of any commercial or financial relationships that could be construed as a potential conflict of interest.

## References

[1] Thery C Zitvogel L Amigorena S Exosomes: composition, biogenesis and function. Nat Rev Immunol (2002) 2(8):569–79 10.1038/nri855 12154376

[2] Théry C Regnault A Garin J Wolfers J Zitvogel L Ricciardi-Castagnoli P Molecular characterization of dendritic cell-derived exosomes. Selective accumulation of the heat shock protein hsc73. J Cell Biol (1999) 147(3):599–610. 10.1083/jcb.147.3.599 10545503 PMC2151184

[3] Bobrie A Colombo M Raposo G Théry C. Exosome secretion: molecular mechanisms and roles in immune responses. Traffic (2011) 12:1659–68. 10.1111/j.1600-0854.2011.01225.x 21645191

[4] Ostrowski M Carmo NB Krumeich S Fanget I Raposo G Savina A Rab27a and Rab27b control different steps of the exosome secretion pathway. Nat Cell Biol (2010) 12:19–30. 10.1038/ncb2000 19966785

[5] Gutiérrez-Vázquez C Villarroya-Beltri C Mittelbrunn M Sánchez-Madrid F. Transfer of extracellular vesicles during immune cell-cell interactions. Immunol Rev (2013) 251:125–42. 10.1111/imr.12013 23278745 PMC3740495

[6] Théry C Ostrowski M Segura E. Membrane vesicles as conveyors of immune responses. Nat Rev Immunol (2009) 9:581–93. 10.1038/nri2567 19498381

[7] Colombo M Raposo G Thery C. Biogenesis, secretion, and intercellular interactions of exosomes and other extracellular vesicles. Annu Rev Cell Dev Biol (2014) 30:255–89. 10.1146/annurev-cellbio-101512-122326 25288114

[8] Valadi H Ekström K Bossios A Sjöstrand M Lee JJ Lötvall JO. Exosome-mediated transfer of mRNAs and microRNAs is a novel mechanism of genetic exchange between cells. Nat Cell Biol (2007) 9(6):654–9. 10.1038/ncb1596 17486113

[9] Théry C Duban L Segura E Véron P Lantz O Amigorena S. Indirect activation of naive CD4+ T cells by dendritic cell-derived exosomes. Nat Immunol (2002) 3(12):1156–62. 10.1038/ni854 12426563

[10] Segura E Amigorena S Thery C. Mature dendritic cells secrete exosomes with strong ability to induce antigen-specific effector immune responses. Blood Cells Mol Dis (2005) 35(2):89–93. 10.1016/j.bcmd.2005.05.003 15990342

[11] Zhang H Xie Y Li W Chibbar R Xiong S Xiang J. CD4+ T cell-released exosomes inhibit CD8+ cytotoxic T-lymphocyte responses and antitumor immunity. Cell and Mol Immunol. (2011) 8:23–30. 10.1038/cmi.2010.59 21200381 PMC4002994

[12] Xie Y Zhang H Li W Deng Y Munegowda MA Chibbar R Dendritic cells recruit T cell exosomes via exosomal LFA-1 leading to inhibition of CD8+ CTL responses through downregulation of peptide/MHC class I and Fas ligand-mediated cytotoxicity. J Immunol (2010) 185:5268–78. 10.4049/jimmunol.1000386 20881190

[13] Busch A Quast T Keller S Kolanus W Knolle P Altevogt P Transfer of T cell surface molecules to dendritic cells upon CD4+ T cell priming involves two distinct mechanisms. J Immunol (2008) 181:3965–73. 10.4049/jimmunol.181.6.3965 18768851

[14] Mittelbrunn M Gutiérrez-Vázquez C Villarroya-Beltri C González S Sánchez-Cabo F González MÁ Unidirectional transfer of microRNA-loaded exosomes from T cells to antigen-presenting cells. Nat Commun (2011) 2:282. 10.1038/ncomms1285 21505438 PMC3104548

[15] Smyth LA Ratnasothy K Tsang JY Boardman D Warley A Lechler R CD73 expression on extracellular vesicles derived from CD4+ CD25+ Foxp3+ T cells contributes to their regulatory function. Eur J Immunol (2013) 43(9):2430–40. 10.1002/eji.201242909 23749427

[16] Okoye IS Coomes SM Pelly VS Czieso S Papayannopoulos V Tolmachova T MicroRNA-containing T-regulatory-cell-derived exosomes suppress pathogenic T helper 1 cells. Immunity (2014) 41(1):89–103. 10.1016/j.immuni.2014.05.019 25035954 PMC4104030

[17] Bryniarski K Ptak W Jayakumar A Püllmann K Caplan MJ Chairoungdua A Antigen-specific, antibody-coated, exosome-like nanovesicles deliver suppressor T-cell microRNA-150 to effector T cells to inhibit contact sensitivity. J Allergy Clin Immunol (2013) 132(1):170–81. 10.1016/j.jaci.2013.04.048 23727037 PMC4176620

[18] Xie Y Zhang X Zhao T Li W Xiang J. Natural CD8(+)25(+) regulatory T cell-secreted exosomes capable of suppressing cytotoxic T lymphocyte-mediated immunity against B16 melanoma. Biochem Biophys Res Commun (2013) 438(1):152–5. 10.1016/j.bbrc.2013.07.044 23876314

[19] Lappas CM Rieger JM Linden J. A2A adenosine receptor induction inhibits IFN-gamma production in murine CD4+ T cells. J Immunol. (2005) 15:1073–80. 10.4049/jimmunol.174.2.1073 15634932

[20] Deaglio S Dwyer KM Gao W Friedman D Usheva A Erat A Adenosine generation catalyzed by CD39 and CD73 expressed on regulatory T cells mediates immune suppression. J Exp Med (2007) 204:1257–65. 10.1084/jem.20062512 17502665 PMC2118603

[21] Kobie JJ Shah PR Yang L Rebhahn JA Fowell DJ Mosmann TR T regulatory and primed uncommitted CD4 T cells express CD73, which suppresses effector CD4 T cells by converting 5’-adenosine monophosphate to adenosine. J Immunol (2006) 177:6780–6 10.4049/jimmunol.177.10.6780 17082591

[22] Romio M Reinbeck B Bongardt S Hüls S Burghoff S Schrader J. Extracellular purine metabolism and signaling of CD73-derived adenosine in murine Treg and Teff cells. Am J Physiol Cell Physiol (2011) 301:C530–9. 10.1152/ajpcell.00385.2010 21593451

[23] Nolte-’t Hoen EN Wagenaar-Hilbers JP Peters PJ Gadella BM van Eden W Wauben MH. Uptake of membrane molecules from T cells endows antigen-presenting cells with novel functional properties. Eur J Immunol (2004) 34(11):3115–25. 10.1002/eji.200324711 15459903

[24] Villarroya-Beltri C Gutiérrez-Vázquez C Sánchez-Cabo F Pérez-Hernández D Vázquez J Martin-Cofreces N Sumoylated hnRNPA2B1 controls the sorting of miRNAs into exosomes through binding to specific motifs. Nat Commun (2013) 4:2980. 10.1038/ncomms3980 24356509 PMC3905700

[25] Cai Z Yang F Yu L Yu Z Jiang L Wang Q Activated T cell exosomes promote tumor invasion via Fas signaling pathway. J Immunol (2012) 188:5954–61. 10.4049/jimmunol.1103466 22573809

[26] Kim SH Bianco N Menon R Lechman ER Shufesky WJ Morelli AE Exosomes derived from genetically modified DC expressing FasL are anti-inflammatory and immunosuppressive. Mol Ther (2006) 13(2):289–300. 10.1016/j.ymthe.2005.09.015 16275099

[27] Klinker MW Lizzio V Reed TJ Fox DA Lundy SK. Human B cell-derived lymphoblastoid cell lines constitutively produce fas ligand and secrete MHCII(+)FasL(+) killer exosomes. Front Immunol (2014) 5:144. 10.3389/fimmu.2014.00144 24765093 PMC3980107

[28] Weiss EM Schmidt A Vobis D Garbi N Lahl K Mayer CT Foxp3-mediated suppression of CD95L expression confers resistance to activation-induced cell death in regulatory T cells. J Immunol (2011) 187(4):1684–91. 10.4049/jimmunol.1002321 21746966

[29] Horwitz DA Pan S Ou JN Wang J Chen M Gray JD Therapeutic polyclonal human CD8+ CD25+ Fox3+ TNFR2+ PD-L1+ regulatory cells induced ex-vivo. Clin Immunol (2013) 149(3):450–63. 10.1016/j.clim.2013.08.007 24211847 PMC3941976

[30] Lechner O Lauber J Franzke A Sarukhan A von Boehmer H Buer J. Fingerprints of anergic T cells. Curr Biol (2001) 11(8):587–95. 10.1016/S0960-9822(01)00160-9 11369203

[31] Raimondi G Shufesky WJ Tokita D Morelli AE Thomson AW. Regulated compartmentalization of programmed cell death-1 discriminates CD4+CD25+ resting regulatory T cells from activated T cells. J Immunol (2006) 176(5):2808–16. 10.4049/jimmunol.176.5.2808 16493037

[32] Mokarizadeh A Delirezh N Morshedi A Mosayebi G Farshid AA Mardani K. Microvesicles derived from mesenchymal stem cells: potent organelles for induction of tolerogenic signaling. Immunol Lett (2012) 147(1–2):47–54. 10.1016/j.imlet.2012.06.001 22705267

[33] Buzas EI György B Nagy G Falus A Gay S. Emerging role of extracellular vesicles in inflammatory diseases. Nat Rev Rheumatol (2014) 10(6):356–64. 10.1038/nrrheum.2014.19 24535546

[34] Garín MI Chu CC Golshayan D Cernuda-Morollón E Wait R Lechler RI. Galectin-1: a key effector of regulation mediated by CD4+CD25+ T cells. Blood (2007) 109(5):2058–65. 10.1182/blood-2006-04-016451 17110462

[35] Vignali DA Collison LW Workman CJ How regulatory T cells work. Nat Rev Immunol (2009) 8:523–32 10.1038/nri2343 18566595 PMC2665249

[36] Kim SH Lechman ER Bianco N Menon R Keravala A Nash J Exosomes derived from IL-10-treated dendritic cells can suppress inflammation and collagen-induced arthritis. J Immunol (2005) 174(10):6440–8. 10.4049/jimmunol.174.10.6440 15879146

[37] Cai Z Zhang W Yang F Yu L Yu Z Pan J Immunosuppressive exosomes from TGF-beta1 gene-modified dendritic cells attenuate Th17-mediated inflammatory autoimmune disease by inducing regulatory T cells. Cell Res (2012) 22(3):607–10 10.1038/cr.2011.196 22157651 PMC3292292

[38] Hall BM Pearce NW Gurley KE Dorsch SE. Specific unresponsiveness in rats with prolonged cardiac allograft survival after treatment with cyclosporine. III. Further characterization of the CD4+ suppressor cell and its mechanisms of action. J Exp Med (1990) 171(1):141–57. 10.1084/jem.171.1.141 2136906 PMC2187663

[39] Tsang JY Tanriver Y Jiang S Xue SA Ratnasothy K Chen D Conferring indirect allospecificity on CD4+CD25+ Tregs by TCR gene transfer favors transplantation tolerance in mice. J Clin Invest. (2008) 118:3619–28. 10.1172/JCI33185 18846251 PMC2564608

[40] Tang Q Lee K. Regulatory T-cell therapy for transplantation: how many cells do we need? Curr Opin Organ Transplant (2012) 17(4):349–54. 10.1097/MOT.0b013e328355a992 22790069

[41] Lee K Nguyen V Lee KM Kang SM Tang Q. Attenuation of donor-reactive T cells allows effective control of allograft rejection using regulatory T cell therapy. Am J Transplant (2014) 14(1):27–38. 10.1111/ajt.12509 24354870 PMC5262439

[42] Xiao F Ma L Zhao M Huang G Mirenda V Dorling A Ex vivo expanded human regulatory T cells delay islet allograft rejection via inhibiting islet-derived monocyte chemoattractant protein-1 production in CD34+ stem cells-reconstituted NOD-scid IL2rgammanull mice. PLoS One (2014) 9(3):e90387 10.1371/journal.pone.0090387 24594640 PMC3940883

[43] Sagoo P Ali N Garg G Nestle FO Lechler RI Lombardi G. Human regulatory T cells with alloantigen specificity are more potent inhibitors of alloimmune skin graft damage than polyclonal regulatory T cells. Sci Transl Med (2011) 3(83):83ra42. 10.1126/scitranslmed.3002076 21593402 PMC3776382

[44] Xiao D Ohlendorf J Chen Y Taylor DD Rai SN Waigel S Identifying mRNA, microRNA and protein profiles of melanoma exosomes. PLoS One (2012) 7(10):e46874. 10.1371/journal.pone.0046874 23056502 PMC3467276

[45] Di Ianni M Falzetti F Carotti A Terenzi A Castellino F Bonifacio E Tregs prevent GVHD and promote immune reconstitution in HLA-haploidentical transplantation. Blood (2011) 117(14):3921–8. 10.1182/blood-2010-10-311894 21292771

[46] Trzonkowski P Bieniaszewska M Juscinska J Dobyszuk A Krzystyniak A Marek N First-in-man clinical results of the treatment of patients with graft versus host disease with human ex vivo expanded CD4+CD25+CD127- T regulatory cells. Clin Immunol (2009) 133(1):22–6. 10.1016/j.clim.2009.06.001 19559653

[47] Brunstein CG Miller JS Cao Q McKenna DH Hippen KL Curtsinger J Infusion of ex vivo expanded T regulatory cells in adults transplanted with umbilical cord blood: safety profile and detection kinetics. Blood (2011) 117(3):1061–70. 10.1182/blood-2010-07-293795 20952687 PMC3035067

[48] Marek-Trzonkowska N Mysliwec M Siebert J Trzonkowski P. Clinical application of regulatory T cells in type 1 diabetes. Pediatr Diabetes (2013) 14(5):322–32. 10.1111/pedi.12029 23627860

[49] Edozie FC Nova-Lamperti EA Povoleri GA Scottà C John S Lombardi G Regulatory T-cell therapy in the induction of transplant tolerance: the issue of subpopulations. Transplantation (2014) 98(4):370–79. 10.1097/TP.0000000000000243 24933458

[50] Zhou X Bailey-Bucktrout SL Jeker LT Penaranda C Martínez-Llordella M Ashby M Instability of the transcription factor Foxp3 leads to the generation of pathogenic memory T cells in vivo. Nat Immunol (2009) 10:1000–7. 10.1038/ni.1774 19633673 PMC2729804

[51] Waldmann H Hilbrands R Howie D Cobbold S. Harnessing FOXP3+ regulatory T cells for transplantation tolerance. J Clin Invest (2014) 124(4):1439–45. 10.1172/JCI67226 24691478 PMC3973097

[52] Yu X Huang C Song B Xiao Y Fang M Feng J CD4+CD25+ regulatory T cells-derived exosomes prolonged kidney allograft survival in a rat model. Cell Immunol (2013) 285(1–2):62–8. 10.1016/j.cellimm.2013.06.010 24095986

[53] Scottà C Esposito M Fazekasova H Fanelli G Edozie FC Ali N Differential effects of rapamycin and retinoic acid on expansion, stability and suppressive qualities of human CD4(+)CD25(+)FOXP3(+) T regulatory cell subpopulations. Haematologica (2013) 98(8):1291–9. 10.3324/haematol.2012.074088 23242600 PMC3729911

[54] Blanchard N Lankar D Faure F Regnault A Dumont C Raposo G TCR activation of human T cells induces the production of exosomes bearing the TCR/CD3/zeta complex. J Immunol (2002) 168(7):3235–41. 10.4049/jimmunol.168.7.3235 11907077

[55] Wahlgren J Karlson Tde L Glader P Telemo E Valadi H. Activated human T cells secrete exosomes that participate in IL-2 mediated immune response signaling. PLoS One (2012) 7(11):e49723. 10.1371/journal.pone.0049723 23166755 PMC3500321

[56] Esposito L Hunter KM Clark J Rainbow DB Stevens H Denesha J Investigation of soluble and transmembrane CTLA-4 isoforms in serum and microvesicles. J Immunol (2014) 193(2):889–900. 10.4049/jimmunol.1303389 24928993 PMC4082723

[57] Pitt JM Charrier M Viaud S André F Besse B Chaput N Dendritic cell-derived exosomes as immunotherapies in the fight against cancer. J Immunol (2014) 193(3):1006–11. 10.4049/jimmunol.1400703 25049431

[58] Escudier B Dorval T Chaput N André F Caby MP Novault S Vaccination of metastatic melanoma patients with autologous dendritic cell (DC) derived-exosomes: results of the first phase I clinical trial. J Transl Med (2005) 3(1):10. 10.1186/1479-5876-3-10 15740633 PMC554765

[59] Morse MA Garst J Osada T Khan S Hobeika A Clay TM A phase I study of dexosome immunotherapy in patients with advanced non-small cell lung cancer. J Transl Med (2005) 3(1):9. 10.1186/1479-5876-3-9 15723705 PMC551593

[60] Delcayre A Le Pecq JB. Exosomes as novel therapeutic nanodevices. Curr Opin Mol Ther (2006) 8(1):31–8. 16506523

[61] Viaud S Théry C Ploix S Tursz T Lapierre V Lantz O Dendritic cell-derived exosomes for cancer immunotherapy: what’s next? Cancer Res (2010) 70(4):1281–5. 10.1158/0008-5472.CAN-09-3276 20145139

[62] Dai S Wei D Wu Z Zhou X Wei X Huang H Phase I clinical trial of autologous ascites-derived exosomes combined with GM-CSF for colorectal cancer. Mol Ther (2008) 16(4):782–90. 10.1038/mt.2008.1 18362931 PMC7106337

[63] Raposo G Stoorvogel W. Extracellular vesicles: exosomes, microvesicles, and friends. J Cell Biol (2013) 200(4):373–83. 10.1083/jcb.201211138 23420871 PMC3575529

[64] Lamparski HG Metha-Damani A Yao JY Patel S Hsu DH Ruegg C Production and characterization of clinical grade exosomes derived from dendritic cells. J Immunol Methods (2002) 270(2):211–26. 10.1016/S0022-1759(02)00330-7 12379326

[65] Théry C Boussac M Véron P Ricciardi-Castagnoli P Raposo G Garin J Proteomic analysis of dendritic cell-derived exosomes: a secreted subcellular compartment distinct from apoptotic vesicles. J Immunol (2001) 166(12):7309–18. 10.4049/jimmunol.166.12.7309 11390481

[66] Yang C Kim SH Bianco NR Robbins PD. Tumor-derived exosomes confer antigen-specific immunosuppression in a murine delayed-type hypersensitivity model. PLoS One (2011) 6(8):e22517. 10.1371/journal.pone.0022517 21829629 PMC3149056

[67] Saunderson SC Dunn AC Crocker PR McLellan AD. CD169 mediates the capture of exosomes in spleen and lymph node. Blood (2014) 123(2):208–16. 10.1182/blood-2013-03-489732 24255917 PMC3888287

[68] Caby MP Lankar D Vincendeau-Scherrer C Raposo G Bonnerot C. Exosomal-like vesicles are present in human blood plasma. Int Immunol (2005) 17(7):879–87. 10.1093/intimm/dxh267 15908444

[69] Wang D Sun W. Urinary extracellular microvesicles: isolation methods and prospects for urinary proteome. Proteomics (2014) 14(6):1922–32. 10.1002/pmic.201300371 24962155

[70] de Candia P Torri A Gorletta T Fedeli M Bulgheroni E Cheroni C Intracellular modulation, extracellular disposal and serum increase of MiR-150 mark lymphocyte activation. PLoS One (2013) 8(9):e75348. 10.1371/journal.pone.0075348 24205408 PMC3805464

[71] Taylor DD Gercel-Taylor C. MicroRNA signatures of tumor-derived exosomes as diagnostic biomarkers of ovarian cancer. Gynecol Oncol (2008) 110(1):13–21. 10.1016/j.ygyno.2008.04.033 18589210

[72] Aushev VN Zborovskaya IB Laktionov KK Girard N Cros MP Herceg Z Comparisons of microRNA patterns in plasma before and after tumor removal reveal new biomarkers of lung squamous cell carcinoma. PLoS One (2013) 8(10):e78649. 10.1371/journal.pone.0078649 24130905 PMC3793941

[73] Ogata-Kawata H Izumiya M Kurioka D Honma Y Yamada Y Furuta K Circulating exosomal microRNAs as biomarkers of colon cancer. PLoS One (2014) 9(4):e92921. 10.1371/journal.pone.0092921 24705249 PMC3976275

[74] Crescitelli R Lässer C Szabó TG Kittel A Eldh M Dianzani I Distinct RNA profiles in subpopulations of extracellular vesicles: apoptotic bodies, microvesicles and exosomes. J Extracell Vesicles (2013) 2:1–10. 10.3402/jev.v2i0.20677 24223256 PMC3823106

[75] Nolte-’t Hoen EN Buermans HP Waasdorp M Stoorvogel W Wauben MH ‘t Hoen PA. Deep sequencing of RNA from immune cell-derived vesicles uncovers the selective incorporation of small non-coding RNA biotypes with potential regulatory functions. Nucleic Acids Res (2012) 40(18):9272–85. 10.1093/nar/gks658 22821563 PMC3467056

[76] Villarroya-Beltri C Baixauli F Gutiérrez-Vázquez C Sánchez-Madrid F Mittelbrunn M. Sorting it out: regulation of exosome loading. Semin Cancer Biol (2014) 28:3–13. 10.1016/j.semcancer.2014.04.009 24769058 PMC4640178

[77] Rossi RL Rossetti G Wenandy L Curti S Ripamonti A Bonnal RJ Distinct microRNA signatures in human lymphocyte subsets and enforcement of the naive state in CD4+ T cells by the microRNA miR-125b. Nat Immunol (2011) 12(8):796–803. 10.1038/ni.2057 21706005

[78] de Candia P Torri A Pagani M Abrignani S. Serum microRNAs as biomarkers of human lymphocyte activation in health and disease. Front Immunol (2014) 5:43. 10.3389/fimmu.2014.00043 24575093 PMC3918657

[79] Lv LL Cao YH Pan MM Liu H Tang RN Ma KL CD2AP mRNA in urinary exosome as biomarker of kidney disease. Clin Chim Acta (2014) 428:26–31. 10.1016/j.cca.2013.10.003 24144866

[80] Oosthuyzen W Sime NE Ivy JR Turtle EJ Street JM Pound J Quantification of human urinary exosomes by nanoparticle tracking analysis. J Physiol (2013) 591(Pt 23):5833–42. 10.1113/jphysiol.2013.264069 24060994 PMC3872755

[81] Henderson MC Azorsa DO. The genomic and proteomic content of cancer cell-derived exosomes. Front Oncol (2012) 2:38. 10.3389/fonc.2012.00038 22649786 PMC3355967

[82] Tolosa JM Schjenken JE Clifton VL Vargas A Barbeau B Lowry P The endogenous retroviral envelope protein syncytin-1 inhibits LPS/PHA-stimulated cytokine responses in human blood and is sorted into placental exosomes. Placenta (2012) 33(11):933–41. 10.1016/j.placenta.2012.08.004 22999499

[83] Harris VK Sadiq SA Biomarkers of therapeutic response in multiple sclerosis: current status. Mol Diagn Ther (2014). 10.1007/s40291-014-0117-0 PMC4245485 25164543

